# Pediatric Acute Lymphoblastic Leukemia Emerging Therapies—From Pathway to Target

**DOI:** 10.3390/ijms24054661

**Published:** 2023-02-28

**Authors:** Anca Viorica Ivanov, Mirabela Smaranda Alecsa, Roxana Popescu, Magdalena Iuliana Starcea, Adriana Maria Mocanu, Cristina Rusu, Ingrith Crenguta Miron

**Affiliations:** 1 Pediatrics Department, Grigore T. Popa University of Medicine and Pharmacy, 16 Universitatii Street, 700115 Iasi, Romania; 2 Medical Genetics Department, Grigore T. Popa University of Medicine and Pharmacy, 16 Universitatii Street, 700115 Iasi, Romania

**Keywords:** acute lymphoblastic leukemia, target therapy, immunotherapy, pediatric

## Abstract

Over the past 40 years, the 5-years-overall survival rate of pediatric cancer reached 75–80%, and for acute lymphoblastic leukemia (ALL), exceeded 90%. Leukemia continues to be a major cause of mortality and morbidity for specific patient populations, including infants, adolescents, and patients with high-risk genetic abnormalities. The future of leukemia treatment needs to count better on molecular therapies as well as immune and cellular therapy. Advances in the scientific interface have led naturally to advances in the treatment of childhood cancer. These discoveries have involved the recognition of the importance of chromosomal abnormalities, the amplification of the oncogenes, the aberration of tumor suppressor genes, as well as the dysregulation of cellular signaling and cell cycle control. Lately, novel therapies that have already proven efficient on relapsed/refractory ALL in adults are being evaluated in clinical trials for young patients. Tirosine kinase inhibitors are, by now, part of the standardized treatment of Ph+ALL pediatric patients, and Blinatumomab, with promising results in clinical trials, received both FDA and EMA approval for use in children. Moreover, other targeted therapies such as aurora-kinase inhibitors, MEK-inhibitors, and proteasome-inhibitors are involved in clinical trials that include pediatric patients. This is an overview of the novel leukemia therapies that have been developed starting from the molecular discoveries and those that have been applied in pediatric populations.

## 1. Introduction

The overall survival (OS) rates in childhood cancers have approached percentages of 75–80% over the past 40 years and specifically for acute lymphoblastic leukemia (ALL) they may attain 90% as a five-year overall survival [1]. Still, despite these significant improvements in survival rates, leukemia remains a major cause of morbidity and mortality, particularly for special subgroups of patients such as infants, adolescents, and patients with high-risk genomic alterations. The chemotherapy, even though it has predictable results given the trials that were performed from the 1960s, also affects the long-term survival due to the adverse effects [1,2,3].

With the potential for less toxicity than current treatment regimens and improved remission outcomes, targeted therapies may provide a new class of oncolytic medications for the treatment of refractory disease or even conventional techniques [3]. The future of leukemia treatment needs to rely more on molecular therapies as well as immune and cellular therapy approaches as the result of thorough risk stratification. It is possible to reduce treatment this way for children that seem to have negative minimal residual disease in the early stages of induction treatment (ex: patients with *ETV6::RUNX1* translocation or hyperdiploid ALL). On the other hand, patients presenting high-risk mutations that are prone to relapse (Philadelphia chromosome (Ph)-positive or Ph-like ALL with *ABL*-class fusion) need more intensified treatment or even the introduction of new therapies that are being tested in numerous trials. Other high-risk subtypes such as hypodiploid ALL, early T-cell precursors, immature T-cells, *KMT2A* rearranged, Ph-positive, and *TCF::HLF*-positive ALL may benefit from treatment with the BCL-2 inhibitor venetoclax. For patients with high-risk leukemia (poor responders in early induction) who do not express genetic alterations that benefit from targeted drugs, immunotherapy could be the hope for efficient treatment [3,4,5].

Advances in the scientific interface have led naturally to advances in the treatment of childhood cancer. These discoveries have involved the recognition of the importance of chromosomal abnormalities, the amplification of the oncogene, and the aberration of the tumor suppressor gene, as well as the dysregulation of cellular signaling and cell cycle control [2,5,6]. Constant efforts toward better characterization of the genetic profile of ALL patients are being made. There are a lot of studies that have explored the biology of acute lymphoblastic leukemia with the intent of finding targets for a more personalized treatment. In the past ten years, leukemia pathways have been defined more precisely in light of new discoveries (mutations, cell-cycle kinases, receptors, proteins, enzymes). These discoveries have made a better classification of leukemia patients possible, leading to a more precise treatment [2,7].

Acute lymphoblastic leukemia is the most frequent malignancy of childhood, and it has been for many years and is still among the principal preoccupations of pediatric oncologists worldwide. ALL is caused by the abnormal clonal proliferation of lymphoid stem cells and is classified as B-lineage acute lymphoblastic leukemia (B-ALL) or T-cell acute lymphoblastic leukemia (T-ALL) depending on whether it originated from a B-cell or a T-cell. A high percentage of childhood B-ALL and T-ALL cases are characterized by chromosomal alterations [8,9]. T-cell acute lymphoblastic leukemia has a poorer prognosis than B-ALL. Fortunately, B-lineage acute lymphoblastic leukemia accounts for a much higher percentage of ALL cases (80–85%), while T-ALL accounts for only 15–25%. The current clinical protocols in ALL use fusion genes for patient stratification and treatment selection, yet a large percentage of patients are unclassified cytogenetically. There are not enough data to permit personalized treatment due to the fact that these types of mutations cause the development of cancer in about 16% of cases [4,8,9]. Identification of other abnormalities of cellular processes involved in the development of clonal propagation of lymphocytes may aid in better classifying patients who remain unclassified. However, for this type of patient who is identified as being high-risk (cortisone resistant, multiple relapses), but without a targetable genetic profile, immunotherapy remains a suitable solution, as demonstrated by the most recent studies.

**Figure 1:**
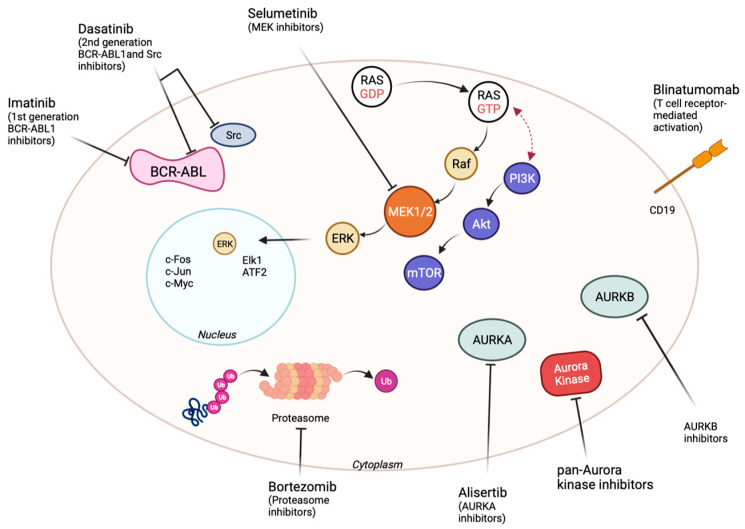
Overview of targetable pathways in pediatric B-ALL.

This is an overview of the novel leukemia therapies that have been developed starting from the molecular discoveries, applied in pediatric populations. Figure 1 provides a graphical summary of the important targets and treatment agents discussed in this review.

## 2. Molecular Targets

### 2.1. Fusion Genes

Fusion genes (FG) are caused by genomic events that include deletions, translocations, insertions, or inversions. These events can be responsible for carcinogenesis through the activation of proto-oncogenes or the inactivation of tumor suppressor genes [10]. Although, over the years, numerous fusion genes have been identified in pediatric ALL patients, the clinical or therapeutic involvement of a large percentage of them is not yet entirely known [4,5]. Acute lymphoblastic leukemia in children is characterized by a number of oncogenic gene fusions which are used as biomarkers for diagnosis, risk assessment, targeted therapy, or prognosis [11]. The number of genetic anomalies in pediatric ALL has increased in recent years as a result of next-generation sequencing technologies, including whole-genome sequencing, whole-exome sequencing, and RNA sequencing [12].

*ETV6::RUNX1*, *TCF3::PBX1*, *BCR::ABL1*, translocations involving the *MLL* gene (*KMT2A::AFF1*, *KMT2A::MLLT3*, *KMT2A::MLLT1*, *KMT2A::MLLT10*), or *PAX5*, among others, are often seen FG in pediatric B-ALL cases [12]. These fusions involve genes that are both oncogenes and crucial for the formation of B cells. The fusion genes involving *DUX4*, *ZNF384*, and *MEF2D* represent important recent findings in pediatric ALL with an impact on the prognosis [13,14,15].

In the T-lineage ALL, FG are reported among 20–30% of patients. The most common modifications are the rearrangements of the T-cell receptor (TCR) genes (*TCRA*, *TCRD*, *TCRB*, and *TCRG*), which activate transcription factor genes such as *TAL* genes (*TAL1*, *TAL2*), *TLX1*, *TLX3*, *LYL1*, *LMO1, cMYC*, *IGH*, and *MLL* [16,17,18].

Various distinct gene abnormalities, such as mutations of the *NOTCH1*, *JAK::STAT*, *PI3K::AKT* pathway genes, and *CDKN2A/2B* deletions, are also found in the pathways controlling differentiation, proliferation, self-renewal, and survival of T-cell progenitors [19]. A fusion oncogene known as *BCR:: ABL1,* was one of the first diagnostic translocations found in patients with chronic myeloid leukemia (CML) in the 1980s. *BCR::ABL1* is a chromosomal fusion between the *BCR* gene from chromosome 22 and the *ABL1* kinase gene from chromosome 9 [20]. Thus, *BCR::ABL* translocations are considered a hallmark of CML [21]. A high-risk subtype of acute lymphoblastic leukemia classified as Philadelphia chromosome ALL is defined by genetic changes that activate cytokine receptor and kinase signaling and is present in 4–5% of pediatric B-ALL cases. The expression of the *BCR::ABL* oncoprotein and the inactivation of the *IKZF1* gene are associated with a poor prognosis in the B-cell precursor cell ALL [22].

Several drugs targeting kinase proteins are approved in the pediatric population. Tyrosine kinase inhibitors (TKIs) are used specifically to treat patients with this translocation, and their survival rates have increased during the past few years in high-income countries. Imatinib mesylate inhibits *ABL1* kinase and was the first drug used in CML patients to improve remission and survival [23]. Depending on whether they detect an active or inactive kinase conformation, TKIs can be classed as type I or type II inhibitors [22]. Depending on when they were first made available for use as a treatment, tyrosine kinase inhibitors can also be categorized into generations: imatinib as first generation TKI [24]. Results from the Children’s Oncology Group study AALL0031 showed that Ph(+) ALL patients treated with chemotherapy and continuous imatinib had 88% 3-year event-free survival [22,25,26]. This shows that the addition of imatinib to initial chemotherapy has a favorable impact on the outcome [27].

Dasatinib and nilotinib are used in cases of imatinib resistance and are both second-generation TKIs. The formation of kinase domain mutations, drug shortages, and the activation of alternative signaling pathways such as the SRC family kinases are frequently linked to resistance mechanisms [24]. Dasatinib is more commonly used as an *ABL/SRC* inhibitor (Figure 1). It plays an important role in imatinib resistance cases crossing the blood-brain barrier [28,29]. On the other hand, current findings demonstrate the superior efficacy of dasatinib as the first-line treatment for Ph+ALL patients, with excellent control of central nervous system leukemia (Table 1) [30].

The first TKI with action against the most prevalent mutation, the *T315I* mutation, is ponatinib, a third-generation TKI. *T315I* is an *ABL*-kinase domain mutation that causes resistance in CML and Ph+ALL [28]. Two phase I/II studies (NCT03934372 and NCT04501614) on ponatinib in pediatric Ph+ALL are ongoing [31,32] (Table 1).

### 2.2. Aurora Kinase Pathway

Aurora kinases are serine/threonine kinases involved in regulating cell cycle progression. They are represented by homologous kinases Aurora A, Aurora B, and Aurora C, which share high-sequence identity [39].

Aurora A is localized to the centrosome or microtubule compartment. It coordinates the maturation of the centrosome, the assembly of the bipolar spindle, and the regulation of the spindle checkpoint, which are necessary for the correct separation of chromosomes in anaphase [40]. In the presence of cofactor Bora, Aurora A regulates G2/M checkpoint release by phosphorylating multiple substrates (*PLK1* of Wee1, *CDC25C*, or direct phosphorylation of *CDK1*) and promotes mitotic entry by controlling CDK1-Cyclin complex activation. In the G2 phase, Aurora-A localizes to pericentriolar material and is activated by the LIM protein Ajuba. It determines the recruitment of several pericentriolar material proteins (such as γ-TuRC, centrosomin, *NDEL1*, *TACC*, *LATS2*, and *BRCA1*) that enhance microtubule nucleation ability and determine centrosome maturation [41]. After the nuclear envelope breakdown, Aurora-A’s role in spindle assembly is exerted at the proximal ends of the microtubule pole. It activates microtubule-associated targeting proteins (such as Ki2a, TACC3, and CKAP5-a) to organize the mitotic spindle, and the interaction with *TPX2* maintains the activation [42].

Between prophase and metaphase, Aurora B is localized to the kinetochores and then migrates during cytokinesis to the cell midbody and cleavage furrow. It provides catalytic activity to the chromosome passenger complex (CPC) consisting of survivin, borealin, and INCENP. Aurora B is involved in mitotic chromosome condensation by phosphorylating chromatin proteins such as histone H3. It contributes to the spindle checkpoint by targeting the microtubule depolymerase mitotic centromere-associated kinesin (MCAK) to kinetochores, regulates the formation of a stable bipolar axe-kinetochore attachment in mitosis, and rectifies erroneous kinetochore attachments to the spindle. Aurora B is necessary for effective cytokinesis at the end of mitosis; without it, the two daughter cells remain joined by cytoplasmic bridges, and the chromosomes misalign, determining the development of binucleate daughter cells [40,43].

The expression of Aurora C is seen in the gonads; however, it is aberrantly expressed in several cancer cell lines. According to reports, it has a similar location to Aurora B and is a chromosomal passenger protein [44].

Several tumor suppressor genes, such as p53, *BRCA1*, *NM23-H1*, and *CHFR*, interact with Aurora-A, and this interaction may be a potent means of promoting carcinogenesis and cell survival [45].

Aurora A overexpression causes centrosome overgrowth, multipolar spindle formation, uneven chromosomal segregation, and aneuploidy, being involved in chromosomal instability and transformation of cancer cells from mitotically active cells [38]. *p53*, *BRCA1/2*, and *RASSF1A* (Ras association domain-containing protein 1) are the major mediators of Aurora A’s participation in aneuploidy [40,43].

Overexpression of Aurora kinases has been found in different human tumors and leukemia cell lines [46]. Aurora B overexpression increases aneuploidy, genomic instability, and the danger of malignant transformation. Cells that overexpress Aurora B stay in mitosis for prolonged lengths of time, most likely due to the suppression of the cyclin-dependent kinase inhibitor p21Cip1. Chromosome segregation, spindle assembly checkpoint activation, maintaining genome integrity, and consequently normal cell division are all compromised by the Aurora B mutation [43,47].

In hematological malignancies (acute myeloid leukemia (AML), myelodysplastic syndrome, chronic myeloid leukemia, ALL), Aurora A and B overexpression are detected in patients with cytogenetic abnormalities that affected to the prognosis and thereby compromise patients’ survival.

In pediatric ALL, overexpression of both Aurora A and B genes has been demonstrated, Aurora B predicting a worse prognosis with significantly lower survival rates being thus more significant compared to Aurora A [48].

It has been demonstrated that AURKB inhibitors compromise cell cycle control checkpoints and spindle formation, disrupt normal chromosome alignment during mitosis, and cause endoreduplication, which causes cells to undergo catastrophic mitosis and apoptosis [41], making this a viable anticancer therapy [42,49].

Aurora kinase inhibitors (AKI) bind to the adenosine 5′- triphosphate (ATP) binding site similar to other small-molecule kinases (for example, imatinib). Aurora kinase inhibitors are either pan-Aurora inhibitors or selective inhibitors for each type, such as Aurora A or Aurora B. Besides the main target, the majority of Aurora kinase inhibitors have the potential to inhibit a domain of “off-target” kinases such as *ABL*, *JAK2*, and *FLT3* to diverse extents [50]. Therefore, being able to inhibit *ABL* and *FLT3*, the most promising hematological use for Aurora kinase inhibitors seem to be in Philadelphia chromosome-positive leukemias and *FLT3*-mutated acute myeloid leukemia, respectively. Inhibition of Aurora A results in the inhibition of autophosphorylation of Aurora A at Thr288, generation of monopolar spindles, G2/M arrest, and apoptosis (Figure 1) [51].

In some types of cancer, studies combined immunotherapy with AKI, and the results were enhanced therapeutic efficacy.

It is important to know the pathways and proteins involved in AURKA-mediated oncogenic function in order to attain the best clinical utility of AKIs. Preclinical studies demonstrated the AKI’s effect on several cellular processes, such as proliferation, invasion, metastasis, autophagy, EMT, chemoresistance, and radioresistance. Additionally, animal studies and clinical studies emphasized the efficiency of AKIs as a single agent or in combination with other standard chemotherapeutic drugs, such as paclitaxel, cisplatin, or combined with targeted therapies [52].

Several studies have showed the efficiency of aurora A—kinase inhibitor (Table 2), Alisertib (MLN8237) in AML cases: NCT02560025 [53], 39 patients, adults, median age 67; under the combination alisertib + citarabine+idarubicine—64% achieved complete remission [54] and NCT01779843 [55], 22 patients, adults, median age 62.7; − alisertib + citarabine+idarubicine, 86% achieved complete remission [56].

All studies were conducted on adult patients with acute myeloblastic leukemia, and the results were satisfactory in those that combined alisertib with chemotherapy. The response rate dropped when the AURKA inhibitor was used alone. Other aurora kinase inhibitors that target both AURKA and AURKB were tested with less encouraging results:

- NCT01380756, 35 AML patients, median age 69; 9% of patients achieved complete remission [57,58];
- NCT01431664, 7 AML+ALL patients with a median age of 3 years; none achieve remission. The current trial represents a first-in-child study of AT9283 in hematological malignancies [59].

In trial NCT01154816, a phase 2 single-arm study including children with relapsed/refractory solid malignancies or acute leukemias, alisertib was evaluated as a potential therapy. The response rate in this study was less than 5% [60,61]. A recent study performed in vitro on cell lines from pediatric patients with ALL showed the efficacy of two AURKA and AURKB designed inhibitors (GW809897X and GW806742X). The experiments performed in this study illustrated cell death induced by both inhibitors through caspase activation and cell cycle arrest. The AURKB expression level was found to be higher than AURKA in this study’s patient samples, sustaining the quality of a suitable biomarker and leading to a promising therapeutic target [59].

### 2.3. MAPK Pathway

Activated ERK pathways regulate cell proliferation, differentiation, apoptosis, and transcription [62].

Four MAPK cascades have been identified: ERK, JNK/stress-activated protein kinase, p38 MAPK, and ERK5 signal transduction pathways. The ERK/MAPK signaling pathway is involved in cell proliferation and differentiation and plays an essential role in the cellular signal transduction network, while the others are mainly correlated with cell stress and apoptosis [63].

Several stimulants, such as growth factors, cytokines, viruses, G-protein-coupled receptor ligands, and oncogenes, activate the ERK pathway. Ras and Raf kinase, as well as MEK1/2 and ERK1/2, are small G proteins that are important players in the ERK/MAPK signaling cascade [64].

RAS regulates signal transduction by changing conformation with GDP (inactive conformation) or GTP (active) binding. Activation is accomplished by multiple factors, such as epidermal growth factor (EGF), tumor necrosis factor, protein kinase C (PKC) activators, and Src family members. Binding of the signal to the receptor leads to the formation of the receptor-Grb2-SOS complex via a Grb2 connector molecule (growth factor receptor-binding protein 2), which binds to the activated receptor with SOS. This causes a high concentration of SOS near Ras, which determines the activation of Ras by changing Ras-GDP to Ras-GTP.

After binding to Ras, the Raf protein, which is encoded by the *RAF* gene, displays serine/threonine protein kinase activity [63].

Ras also produces a signal output through a variety of additional effector pathways, such as PI3K/Akt/mTOR, RalGEF/RAL, and Raf/MEK/ERK. Ras and the PI3K pathway interact often, and this interaction is crucial for the control of cell growth and survival [65].

The Raf kinase family has three subtypes: Raf-1, A-Raf, and B-Raf. In the Ras/Raf/MEK/ERK proliferation signal transduction pathway, Ras, as the upstream activated protein, uses two regions, the Ras-binding domain and the cysteine-rich domain at the N-terminus of Raf-1, to bind and translocate Raf from the cytoplasm to the cell membrane, where Raf is activated. Activated Raf-1 continues to activate downstream MEK and MAPK and finally delivers cell proliferation and differentiation signals to the nucleus by regulating the activity of various transcriptional regulators [63].

Activated MEK catalyzes the bispecific phosphorylation and dimerization of ERK. Activated ERKs are translocated from the cytoplasm to the nucleus and regulate the activity of different transcription factors (proto-oncogene *c-Fos*, proto-oncogene *c-Jun*, ETS domain-containing protein Elk-1, proto-oncogene *c-Myc*, and cAMP-dependent transcription factor ATF2), determine the phosphorylation of some cytoplasmic target proteins (such as microtubule-associated protein MAP 1, 2, and 4 involved in the regulation of cell shape and cytoskeletal redistribution) or regulate the activity of other kinases by phosphorylation of downstream or even upstream of the ERK pathway substrates (SOS, Raf-1 and MEK—negative feedback regulation) [63].

The ERK/MAPK signaling cascade controls apoptosis by upregulating the gene expression of Bcl-2 family members that promote survival and the selection of anti-apoptotic proteins for proteasomal degradation [65].

Numerous malignancies commonly exhibit constitutive activation of this system, which has been proven to give cancer cells a proliferative edge and malignant phenotypes [66].

Frequently, in ALL (with a predominance of B lineage), the Ras pathway is activated by somatic mutations of genes that affect regulatory proteins, upstream activators, and pathway components, including *NRAS*, *KRAS*, *BRAF*, *FLT3*, *PTPN11*, *CBL*, and *NF1* [55]. Ras pathway mutations are common in cytogenetic subgroups with high risk and low risk, pointing to a potential connection between chromosomal instability and dysregulated MAPK-ERK pathway signaling [67,68].

Constitutive activation of the Ras pathway is found in low-risk chromosomal translocations, including *BCR::ABL* and those involving the *MLL* locus, and is rare in the ETV6-*RUNX1* cytogenetic subgroup [69].

In infants with gene fusions with *MLL*, *NRAS/KRAS* mutations are present in up to half of the cases and have been shown to be an independent prognostic factor associated with unfavorable evolution [65].

In pediatric T-ALL samples, the MAPK-ERK pathway is aberrantly activated by mutant and physiological *IL7R* signaling. BIM (a pro-apoptotic BCL2 family protein) can bind to anti-apoptotic family members, including BCL2, BCLW, BCLXL, and MCL1, to block their cell survival activities. Activated MEK-ERK phosphorylates pro-apoptotic BIM, which thereby impairs its binding to anti-apoptotic proteins, causing MAPK-ERK-induced steroid resistance [70]. Additionally, by upregulating p53, suppression of the MAPK-ERK signaling cascade in ALL may improve steroid sensitivity [71].

It was proven that circ RNA Circ-0000745 induced the ERK signaling pathway, which increased ALL cell proliferation [72].

Several studies have showed that mutations in genes that activate the Ras/Raf/Mek/Erk pathway (*NRAS*, *KRAS*, *FLT3*, and *PTPN11*) are encountered in relapsed ALL and seem to respond to the MEK inhibitor selumetinib (Figure 1).

Selumetinib is a selective inhibitor of MEK1/2 with anti-tumor activity demonstrated by several studies (phase III clinical trials for a few types of adult solid tumors) and manageable toxicity. The phase I clinical trial for children with recurrent or refractory low-grade glioma (with *BRAF* mutation) is the most recent trial on the pediatric population treated with selumetinib [73].

In vitro studies have shown that patients with relapsed cancer have significant cortisol resistance as well as resistance to other chemotherapies [73,74].

Selumetinib and glucocorticoids share the proapoptotic protein—BIM (Bcl-2 protein 11) for the induction of cell death, a fact that leads to the hypothesis that working together could improve therapeutic effect. In vitro experiments in RAS pathway-mutated acute lymphoblastic leukemia cells indicated a strong synergism (combination index <0.2; n = 5). Moreover, the pharmacodynamic assays confirmed this hypothesis [75].

An international phase I/II clinical trial of dexamethasone and selumetinib (Seludex trial) is underway in children with multiply relapsed/refractory disease (NCT03705507), a phase I/II trial in children with r/r RAS pathway mutations in ALL [76].

### 2.4. The Ubiquitin-Proteasomal System (UPS)

The ubiquitin-dependent proteasome is a system made up of six components: ubiquitin, ubiquitin-activating enzymes (UBA, E1), ubiquitin-conjugating enzymes (UBC, E2), ubiquitin ligases (E3), the proteasome, and deubiquitinases (Dub).

Ubiquitin is a small protein made up of 76 amino acids that bind covalently to a specific substrate protein. Among the 76 amino acids of ubiquitin, there are seven lysine residues (K6, K11, K27, K29, K33, K48, and K63), which are the bridge to the target proteins. Ubiquitin is linked to the substrate through the E1-E2-E3 cascade [77,78], a process called protein ubiquitination, and can be removed by Dubs if necessary [79].

Ubiquitination is one of the most important systems for regulating protein function and is involved in cell metabolism, cell proliferation, glycogen synthesis, cell death, and in diseases such as inflammation, arthritis, heart diseases, and cancer [80].

Once a protein is ubiquitinated, it will change its structural conformation, cellular localization, and biological function, or it will be degraded by specific proteases in the 26S proteasome complex [77,81,82,83].

Proteasome 26S is a large protein complex, composed of one 20S core particle and two 19S regulatory particles. The 20S particle is made up of two α units (at the two ends) and two β units (in the middle). Each of these units is composed of 7 subunits, and the 28 subunits stack to form a cylinder [82,84].

Proteases are found only on the inner surface of β subunits, specifically β1, β2, and β5 with chymotrypsin-like, trypsin-like, and peptidyl-glutamyl peptide-hydrolyzing (PHGH) activity, respectively [81].

Bortezomib is a reversible, selective inhibitor of the 26S proteasome and it is considered a targeted therapy in several oncologic diseases (Figure 1). Bortezomib was initially approved by the Food and Drug Administration for the treatment of multiple myeloma and relapsed non-Hodgkin lymphoma. Several studies have shown that proteasome number and activity are increased in hematologic malignancies, including AML and ALL (NCT00873093) [85,86].

Bortezomib has a proapoptotic effect on plasma cells and the bone marrow microenvironment by inhibiting the ubiquitin-proteasome pathway, which plays a prominent role in regulating cell growth, both in normal and neoplastic cells. Angiogenesis, cell-cell adhesion, and proliferation, processes related to the ubiquitin-proteasome pathway, may also be inhibited by the use of Bortezomib [87]. The administration of Bortezomib can be via the intravenous or subcutaneous route with a rapid initial distribution and large volume of distribution, but the drug does not pass the blood or brain barrier. Before administration, hepatic function must be assessed because hepatic impairment causes high plasma levels of bortezomib with severe side effects [88].

## 3. High-Risk Leukemia Patients without Molecular Targets

A significant proportion of high-risk ALL patients with poor response to early induction treatment do not express targetable genetic alterations, nor do they have reachable molecular therapies. For this type of patient, the treatment solutions currently available would be, leaving aside intensive chemotherapy, the treatments that give a lot of hope: immunotherapy and adoptive cell therapy.

Blinatumomab, a bispecific T-cell engager antibody, is an important representative of this class of drugs. The mechanism of action consists of binding to CD3 on the surface of T cells and CD19 on leukemia cells, initiating T cell receptor-mediated activation, and killing CD19-positive B-ALL (Figure 1) [89,90]. In several trials of individuals with refractory, recurrent, and newly diagnosed Philadelphia chromosome-negative or positive ALL, it has been demonstrated to enhance prognosis (Table 3) [90,91].

In fact, patients with Philadelphia chromosome-positive ALL may benefit from combining an ABL tyrosine kinase inhibitor and blinatumomab. After a phase I/II trial with a 39% complete remission rate and 52% of the responders obtaining an minimal residual disease (MRD) negative status during the first two cycles of therapy, blinatumomab was recently licensed for pediatric patients with relapsed or refractory ALL [89,93,97]. Although blinatumomab is often well tolerated, it has been linked to serious and even fatal side effects, such as neurotoxicity and cytokine release syndrome, which can happen concurrently or separately. A debulking sequential combination method may help to lower the incidence of cytokine release syndrome [98,99,100]. In order to increase T cell activation and subsequently the action of the antibody, immune checkpoint inhibition and blinatumomab therapy are being investigated in adults (NCT03160079) [99,101].

A number of studies using blinatumomab for relapsed patients included children:

- NCT02807883—completed, 23 patients were enrolled, the 1-year overall survival was 85%, progression-free survival (PFS)—71%, and nonrelapse mortality (NRM) rates and 0%, respectively [92];
- NCT01471782—included children and adolescents up to 17 years of age with relapsed/refractory B-cell precursor acute lymphoblastic leukemia (BCP-ALL); 49 patients were treated in phase I and 44 patients in phase II. Among the 70 patients who received the recommended dosage, 27 achieved complete remission within the first two cycles, 14 (52%) of whom achieved a complete minimal residual disease response. This was the first such trial performed on children that evidenced the efficient antileukemic activity of single-agent blinatumomab in achieving a complete MRD response in children with relapsed/refractory BCP-ALL [93];
- NCT04723342—study is still recruiting—the goal of the study is to improve the treatment of patients with primary B-cell precursor acute lymphoblastic leukemia by incorporating monoclonal bispecific antibodies in post-induction treatment while reducing chemotherapy [94];
- NCT02412306—included children and adults. A few serious adverse effects were registered, such as cytokine release syndrome in 1 (6%) pediatric patient (Blinatumomab was discontinued). Eleven (79%) adults achieved complete remission, and also five (29%) pediatric patients, of which two had an MRD response [95];
- NCT02877303—still recruiting; Blinatumomab, Inotuzumab Ozogamicin, and Combination Chemotherapy as Frontline Therapy in Treating Patients With B Acute Lymphoblastic Leukemia—Includes Patients Over 14 Years of age; incorporation of Inotuzumab Ozogamicin, blinatumomab, and venetoclax in frontline ALL [96].

Inotuzumab Ozogamicin (InO) is an anti-CD22 monoclonal antibody linked to the cytotoxic antibiotic calicheamicin [102]. CD22 is strongly expressed on leukemic blasts in more than 90% of pediatric BCP-ALL, making it a perfect target for immunotherapy. Since 2017, InO has been approved for adults with CD22-positive relapsed/refractory BCP-ALL, based on data from the INO-VATE ALL trial (NCT01564784). Patients who received InO reported higher complete remission and complete remission with incomplete hematologic recovery rates (73.8 vs. 35%), a longer median remission (5.4 vs. 4.2 months), and longer progression-free survival (5 vs. 1.7 months) than those receiving chemotherapy [103,104].

The experience with inotuzumab ozogamicin in pediatric patients is limited. A report of five pediatric patients diagnosed with refractory/relapsed B-cell ALL and enrolled in a phase II non-randomized trial of InO showed an achievement of complete remission (CR) or complete remission with incomplete hematologic recovery (CRi) after one or two courses in three patients. All patients underwent hematopoietic stem cell transplantation (HSCT) and relapsed shortly after [105].

Data collected from 51 pediatric patients diagnosed with relapsed/refractory B-ALL who received InO through a compassionate use program showed complete remission or complete remission with incomplete hematologic recovery in 67% of cases (28 patients). In 20 patients, the minimal residual disease was negative, and 21 patients received hematopoietic stem cell transplantation. The event-free survival rate was 23.4 7.5% at 12 months, and the overall survival rate was 36.3 9.3% [105,106].

A single-arm phase II trial (NCT02981628) is being conducted by Children’s Oncology Group on the safety and efficacy of InO in pediatric and adolescent patients with relapsed or refractory B-cell acute lymphoblastic leukemia. Nineteen patients achieved complete remission with incomplete hematologic recovery, three patients had a partial response after the first cycle of InO, and two more patients after the second course of InO.

A multicenter French pediatric retrospective study assessed the efficacy and the toxicities of InO in pediatric patients. Twelve pediatric patients received InO through a compassionate use program. CR/Cri was achieved in eight patients after the first cycle of InO, and only two of them had MRD negativity. With a mean follow-up of 23 months, two of the four patients who underwent HSCT following InO are disease-free. Event free survival rate (EFS) was 33% ± 13·6% and OS 38% ± 14% at 12 months [107].

The main toxicities were represented by hepatotoxicity, infusion-related hypotension, febrile neutropenia, infections, and fever. The sinusoidal obstruction syndrome occurred frequently in HSCT patients [105,106,107].

### CAR T-Cell Therapy

The creation of chimeric antigen receptor T cell (CAR T-cell, CARs) treatment has been a very promising area for immunotherapy in hematologic malignancies. This treatment includes both direct targeting of tumor antigens and enhancement of these specific immune effectors [108]. Chimeric antigen receptor T cells depend on the transfer of genetically altered effector cells to induce an antileukemic immune response. As a result, they have the potential to survive in vivo, providing long-term disease management. CAR T-cells employ a strategy independent of human leukocyte antigen, being modified to produce chimeric antigen receptors directed against a particular tumor surface antigen; as a result, they are both antigen-specific and major histocompatibility complex (MHC)-independent [109]. The basic structure of CARs consists of an antibody-derived single-chain variable fragment joined to an intracellular T cell signaling domain with a costimulatory domain by a hinge and transmembrane domain (number and type depending on the specific CAR) [110,111].

For the production of CARs, autologous T cells are used, but also allogeneic CARs and CAR- NK cells have been created [112,113]. In 2017, the anti-CD19 CAR T-cell therapy Tisagenlecleucel, formerly known as CTL019, was the first B-ALL treatment to obtain Food and Drug Administration (FDA) approval. Usually this type of treatment is licensed for children and young adults after approval for adults but CAR T-cell proved an outstanding efficacy in this particular age group [114].

Leukapheresis is used to collect autologous T cells, which is the first step in the CAR T-cell therapy process for patients. The CARs are then inserted into the T cells via one of various ways, the most popular of which uses viral vectors, and the cells are then cultured in order to grow. Prior to CAR T-cell infusion, patients typically receive lymphodepleting chemotherapy, which can promote in vivo T cell proliferation by upregulating the expression of homeostatic cytokines including IL-7 and IL-15. This in vivo expansion has been linked to therapeutic response; as a result, it might be more important than the actual dose of T cells given [108,110,115].

The first published report on the use of CAR T-cell in treating B-ALL was in 2013. Two young patients were given 19-BBz CAR T-cells and they experienced full remission, while the CARs multiplied more than 1000 times and were later found in their bone marrow and cerebrospinal fluid for at least six months [116]. Following that, a case series of 30 B-ALL patients, including children and adults, was treated with 19-BBz CAR T cells at a single facility and, 90% of them achieved complete remission [110].

CAR T-cell technology has undergone numerous modifications since being approved and has been used in numerous clinical trials. Although CD19 is still the most often targeted antigen, CAR T-cells against CD22 have also been created. Researchers created second- and third-generation CARs that were modified to contain more costimulatory domains after first-generation CARs underperformed in clinical testing. Because of their increased efficacy and safety, second- and third-generation CARs are used in the majority of ongoing clinical trials.

Most of the trials using CAR T-cell as treatment for relapsed ALL patients (children and young adults) that are completed, published promising results:

- NCT02435849—ELIANA trial (global collaborative study) included 75 patients, children and young adults treated with CAR T-cell (CD19). The overall remission rate (OR) was 81%, with all patients being negative for MRD. The event-free survival and overall survival were 73% and 90%, respectively, at 6 months and 50% and 76%, respectively, at 12 months [114];
- NCT03289455—AMELIA trial—a phase 1 trial of 45 children and young adults with relapsed or refractory B-lineage acute lymphoblastic leukemia was conducted using a CD19 CAR-T. The MRD^-^ remission rate was 93% [117];
- NCT02315612—single center phase 1 trial, included 58 children and young adults treated with CD22 CAR T-cell infusion. The CR was 70%, the median OS was 13.4 months and for the patients who achieved a complete response, the median relapse-free survival was 6.0 months [118];
- NCT01860937—multicenter clinical trial that included 25 patients (children and young adults) treated with CD19 CAR T-cell—75% patients achived CR, 89% were MRD negative [119];
- NCT01593696—phase I trial on children and young adults—50 patients were included, treated with CD19 CAR T-cell (autologus). A total of 62.0% patients achieved a complete remission (CR), 90.3% presented MRD negative [120].

CAR T-cell therapy has eliminated the need for HSCT in certain patients, but it does not stop recurrence because of antigen escape, a lack of CAR T-cell persistence, or T-cell exhaustion. Some patients may still need consolidation with HSCT for definitive therapy, such as those recieving CD22-directed CARs or those with positive MRD identified by next-generation sequencing post-CAR therapy [111].

## 4. Conclusions

Since the introduction of genomic sequencing and other molecular techniques, cancer treatment has held the promise of a new generation of therapies. Using these new technologies, it has been possible to find targets and make more drugs that hit these targets. The goal is to eventually replace conventional chemotherapy with more precise therapy that would increase the number of people who get better and improve their quality of life.

Gene discovery investigations have improved other elements of the precision medicine algorithm, such as cancer surveillance, evaluation of inherited vulnerability to therapy-related toxicity, and treatment response monitoring, in addition to providing new therapeutic targets.

Advances in leukemia genomics have provided new prospects for precision medicine strategies, enhanced our understanding of the biology of the illness, and revealed actionable genomic changes. Trials of molecular-targeted therapy are now being conducted in an effort to balance toxicity and survival for various patient populations with high risk. However, this innovative era of customized therapy also presents new difficulties. The objectives of the following generation of translational trials are to identify the best combination of innovative therapeutic agents and traditional cytotoxic medications and to look into the fundamental processes driving therapy resistance.

We are now witnessing the slow but steady development of these new therapies in the hope of overcoming the difficulties typically encountered by studies involving children: smaller number of participants due to the rarity of the disease; different biology of growing organisms that may influence drug effect; and trial stopped because the adult study did not achieve the end-point. 

## Figures and Tables

**Table 1:** Second and third generation tirozin-kinase inhibitors in pediatric Ph+ALL.

Study Number	Patients	Drug	Results	Reference
NCT01460160	106 pediatric patients Ph+ALL	Dasatinib Standard chemotherapy	53.1% 5-year EFS	[33,34]
NCT00720109	63 participants (53 pediatric and 7 adult patients) Ph+ALL	Dasatinib Standard chemotherapy	60% 5-year EFS 86% 5-year OS	[35,36]
NCT01077544	15 pediatric patients 11 diagnosed with CML/4 diagnosed with Ph+ALL	Nilotinib Standard chemotherapy	Ph+ALL: 75% complete remission, 25% stable disease	[37,38]
NCT04501614	Estimated 60 pediatric patients Ph+ALL/MPAL/Ph-like ALL	Ponatinib	No results/active, not recruiting	[5]
NCT03934372	Estimated 60 pediatric patients CML/ALL/Lymphoma/Solid tumors	Ponatinib	Still recruiting	[32]

Ph+ALL—Philadelphia chromosome positive acute lymphoblastic leukemia; CML—chronic myeloid leukemia; MPAL—Philadelphia chromosome-positive mixed phenotype acute leukemia; EFS—event free survival; OS—overall survival.

**Table 2:** Aurora kinase inhibitors studies.

Study Number	Patients	Drug	Results	References
NCT02560025	39 AML patients, adults, median age 67	Alisertib (+chemotherapy)	65% CR	[53]
NCT01779843	22 AML patients, adults, median age 62.7	Alisertib (+chemotherapy)	86% CR	[55]
NCT01380756	35 AML patients, median age 69	AURKA and AURKB inhibitors	9% CR	[57,58]
NCT01431664	7 AML+ALL patients, median age 3 years	AURKA and AURKB inhibitors	0% R	[59]
NCT01154816	118 children with relapsed/refractory solid malignancies or acute leukemias.	Alisertib	<5% R	[60,61]

ALL—acute lymphoblastic leukemia, AML—Acute myeloblastic leukemia, CR—complete response, R—response.

**Table 3:** Blinatumomab studies.

Study Number	Patients	Drug	Results	Reference
NCT02807883	23 ALL patients (children + adults)	Blinatumomab (following HCT)	OS 85%, PFS 71%, NRM 0%	[92]
NCT01471782	93 ALL patients (children < 17 years)	Blinatumomab	52% CR	[93]
NCT04723342	180 estimated participants	Blinatumomab (post-induction)	Still recruiting	[94]
NCT02412306	66 elapsed/refractory ALL patients (Children + adults)	Blinatumomab (consolidation)	11 adults, 5 children CR	[95]
NCT02877303	80 estimated participants > 14 years	Blinatumomab, Inotuzumab Ozogamicin and Combination Chemotherapy	Still recruiting	[96]

ALL—acute lymphoblastic leukemia, AML—acute myeloblastic leukemia, CR—complete response, NRM—(non-relapse mortality), OS—overall survival.

## Data Availability

Not applicable.
